# ROCK2 Regulates Monocyte Migration and Cell to Cell Adhesion in Vascular Endothelial Cells

**DOI:** 10.3390/ijms20061331

**Published:** 2019-03-16

**Authors:** Yusuke Takeda, Keiichiro Matoba, Daiji Kawanami, Yosuke Nagai, Tomoyo Akamine, Sho Ishizawa, Yasushi Kanazawa, Tamotsu Yokota, Kazunori Utsunomiya

**Affiliations:** Division of Diabetes, Metabolism and Endocrinology, Department of Internal Medicine, Jikei University School of Medicine, 3-25-8 Nishishinbashi, Minato-ku, Tokyo 105-8461, Japan; ms05-takeda@jikei.ac.jp (Y.T.); daijika@jikei.ac.jp (D.K.); y.nagai@jikei.ac.jp (Y.N.); akamine-tm@igakuken.or.jp (T.A.); ishizawa@jikei.ac.jp (S.I.); yasu@jikei.ac.jp (Y.K.); yokotat@jikei.ac.jp (T.Y.); kazu-utsunomiya@jikei.ac.jp (K.U.)

**Keywords:** Rho-kinase, ROCK2, atherosclerosis, endothelial function, inflammation

## Abstract

The small GTPase Rho and its downstream effector, Rho-kinase (ROCK), regulate various cellular functions, including organization of the actin cytoskeleton, cell adhesion and migration. A pro-inflammatory lipid mediator, lysophosphatidic acid (LPA), is a potent activator of the Rho/ROCK signalling pathway and has been shown to induce the expression of chemokines and cell adhesion molecules (CAMs). In the present study, we aimed to elucidate the precise mechanism by which ROCK regulates LPA-induced expressions and functions of chemokines and CAMs. We observed that ROCK blockade reduced LPA-induced phosphorylation of IκBα and inhibited NF-κB RelA/p65 phosphorylation, leading to attenuation of RelA/p65 nuclear translocation. Furthermore, small interfering RNA-mediated ROCK isoform knockdown experiments revealed that LPA induces the expression of monocyte chemoattractant protein-1 (MCP-1) and E-selectin via ROCK2 in human aortic endothelial cells (HAECs). Importantly, we found that ROCK2 but not ROCK1 controls LPA-induced monocytic migration and monocyte adhesion toward endothelial cells. These findings demonstrate that ROCK2 is a key regulator of endothelial inflammation. We conclude that targeting endothelial ROCK2 is potentially effective in attenuation of atherosclerosis.

## 1. Introduction

Atherosclerosis is a chronic arterial disease that is caused by inflammatory and regenerative processes. In the progression of the atherosclerosis, endothelial-leukocyte adhesion molecules including vascular cell adhesion molecule-1 (VCAM-1) and E-selectin play a key role in the early adhesion of mononuclear leukocytes to arterial endothelium at sites of atheroma initiation. Once adherent to the endothelial cell, leukocytes migrate through the endothelium by chemokines [1]. Lysophosphatidic acid (LPA), a pro-inflammatory lipid mediator, is produced and released from activated platelet [2]. Serum levels of LPA are elevated in a variety of pathological settings including diabetes and acute coronary syndrome [2,3,4,5]. LPA accumulates in atherosclerosis lesions [6,7] and it triggers the release of the chemokines including CCLs, CXCLs, colony-stimulating factors and interleukins from endothelial cells through its receptors [8,9,10]. Furthermore, inhibition of LPA receptors has been shown to attenuate atherosclerosis development in LDL receptor-deficient mice [11]. Despite the importance of lipid mediator-induced vascular inflammation, our understanding of the molecular mechanisms that governs atherosclerosis remains incomplete. Given the epidemic of cardiovascular disease, understanding the precise mechanism regulating LPA-induced atherogenic signalling is of considerable interest.

Rho-kinase (ROCK) was initially characterized as a mediator of Rho-induced stress fibre formation [12]. Activation of the Rho/ROCK pathway leads to the phosphorylation of downstream substrates, including myosin phosphatase target subunit [13], which has been postulated to control diverse cellular functions such as cell proliferation, contraction, migration and gene expression [14]. A series of recent studies have demonstrated that Rho/ROCK signalling plays a crucial role in cardiovascular disease [15,16,17]. ROCK has two isoforms, ROCK1 and ROCK2 that share 65% sequence similarity [18]. Interestingly, it has become increasingly clear that ROCK1 and ROCK2 show distinctive roles in regulating endothelial function. For instance, Shimada et al. indicated the contribution of ROCK2 to the induction of intracellular adhesion molecule-1 (ICAM-1) and VCAM-1 through NF-κB activation [2]. However, it remains unclear (1) what other genes are regulated by ROCK, (2) how ROCK regulates NF-κB signalling, (3) whether ROCK2 isoform has specific role in endothelial dysfunction by modulating chemokines and CAMs. We aimed to address these questions in the present study.

## 2. Results

### 2.1. Regulation of Inflammatory Cytokines, Receptors and Adhesion Molecules via ROCK

**Figure 1:**
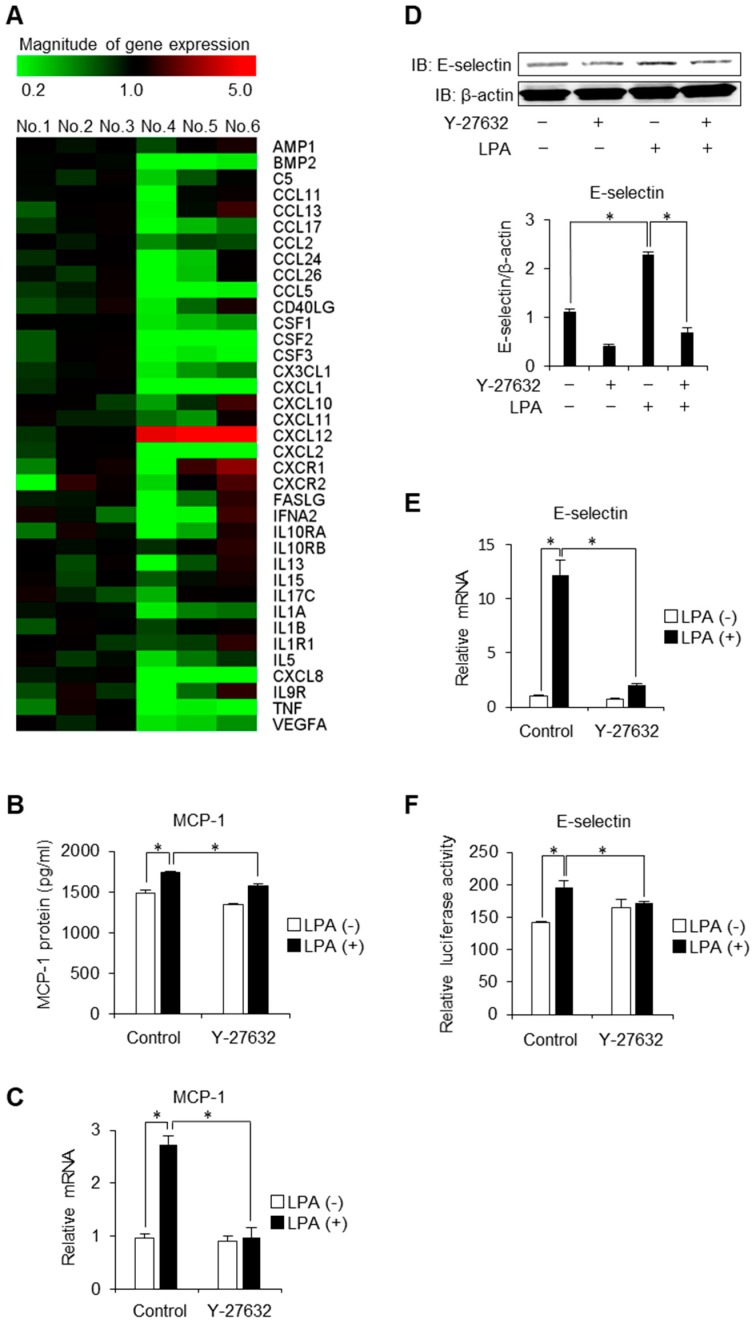
ROCK modulates expression of inflammatory cytokines, receptors and adhesion molecule. (**A**) Inflammatory cytokines and receptors PCR array for HAECs. No.1–3 samples were stimulated with LPA (50 μM) for 8 h. No.4–6 samples were pre-treated with Y-27632 (10 μM) before stimulation with LPA (50 μM) for 8 h. Heat map depicts the relative expression values for the 37 cytokines (*n* = 3). (**B**) HAECs were treated with Y-27632 (10 μM) for 30 min and then were stimulated by LPA (50 μM) for 12 h. Culture media were harvested and followed by ELISA (*n* = 3). * *p* < 0.05. (**C**) HAECs were pre-treated with Y-27632 (10 μM) before stimulation with LPA (50 μM) for 4 h. MCP-1 mRNA was analysed by quantitative real-time PCR (*n* = 3). * *p* < 0.05. (**D**) HAECs were pre-treated with Y-27632 (10 μM) and then stimulated with LPA (50 μM) for 8 h. Cell lysates were subjected to Western blot analysis for E-selectin. β-actin was loaded as internal control. The histogram shows the relative intensity of each band (*n* = 3). * *p* <0.05. (**E**) HAECs were pre-treated with Y-27632 (10 μM) before stimulation with LPA (50 μM) for 8 h. E-selectin mRNA was analysed by quantitative real-time PCR (*n* = 3). * *p* < 0.05. (**F**) HAECs were transfected with a pGL3-ELAM-LUC construct. Cells were pre-treated with Y-27632 (10 μM) before stimulation with LPA (50 μM) for 4 h. The bar graph shows the relative luciferase activity of each sample (*n* = 3). * *p* < 0.05. Data are expressed as means ± SEM.

To comprehensively assess the influence of ROCK on the development of atherosclerosis, we first performed PCR array screening the entire inflammatory pathway. As a result, a large number of chemokines and inflammatory cytokines including CCL, CSF and TNF, which have been demonstrated to be upregulated by LPA [10,19,20], were downregulated by a chemical inhibitor of ROCK (Y-27632) in LPA-stimulated HAECs, indicating a broad effect of ROCK on the inflammatory machinery (Figure 1A). Of note, there was a trend of upregulation in some genes including CXCL10, CXCL11, CXCR1, CXCR2 and CXCL12 was significantly increased by ROCK inhibition.

MCP-1 is a potent monocyte agonist and the absence of MCP-1 provides dramatic protection from macrophage recruitment and atherosclerotic lesion in apo B transgenic mice [21]. Consistently with the results obtained from PCR array, real-time PCR and Western blot analysis demonstrate that Y-27632 inhibits LPA-induced MCP-1 protein secretion and mRNA expression (Figure 1B,C), confirming the contribution of ROCK in the MCP-1 induction.

We next investigated the potential role of ROCK in mediating the induction of E-selectin. E-selectin acts as an adhesion molecule mediating the first step in leukocyte extravasation and plays an important role in the development of coronary heart diseases [22]. As shown in Figure 1D, E-selectin was induced by the stimulation of LPA. This induction was suppressed by Y-27632, indicating that LPA induces E-selectin in a ROCK-dependent manner. Consistently, Y-27632 suppressed mRNA expression and promoter activity of E-selectin (Figure 1E,F). Taken together, these data provide evidence for the broad contribution of ROCK in the pathogenesis of endothelial inflammation.

### 2.2. Phosphorylation of IκBα is Mediated via ROCK Signaling

**Figure 2:**
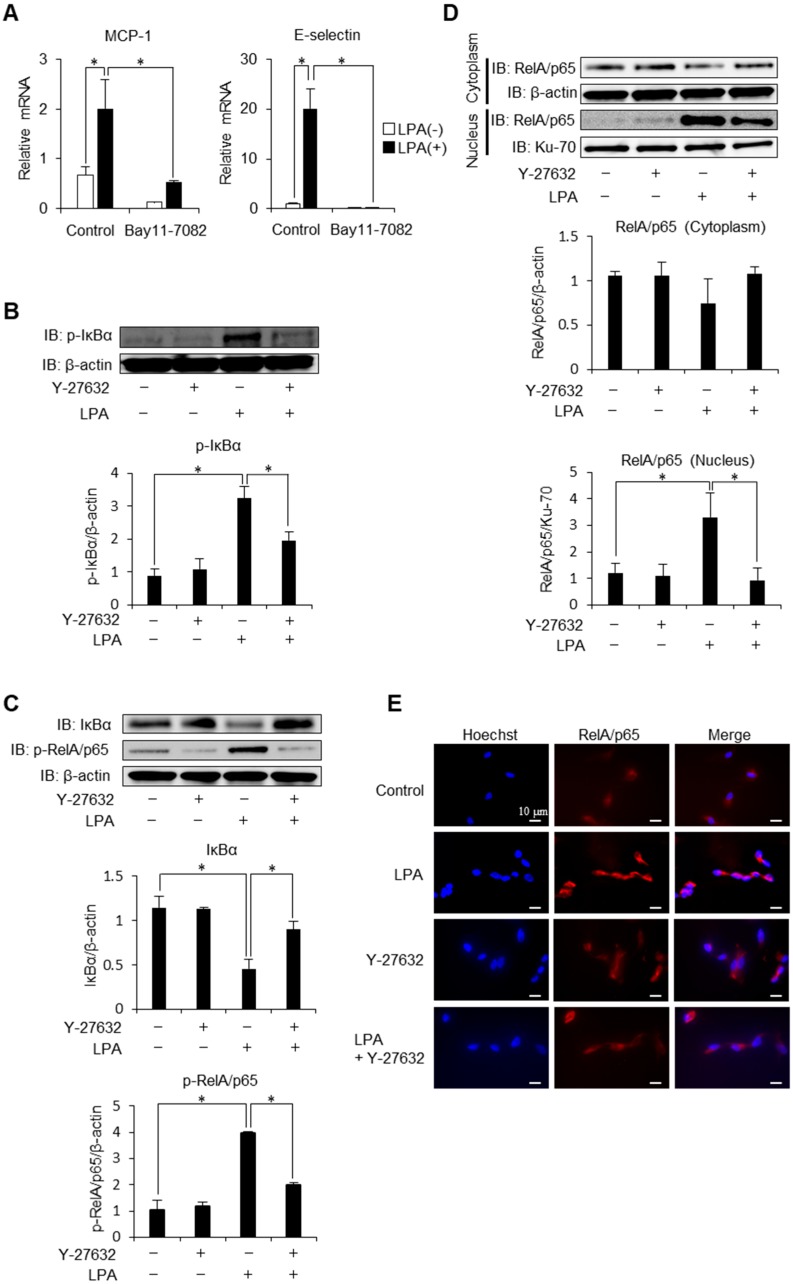
Kinetics of IκBα is under the control of ROCK signalling. (**A**) HAECs were pre-treated with Bay 11-7082 (5 μM) before stimulation with LPA (4 h). MCP-1 and E-selectin mRNA expression levels were analysed by quantitative real-time PCR (*n* = 3). * *p* < 0.05 vs. LPA alone. (**B**) HAECs were pre-treated with Y-27632 (10 μM) before stimulation with LPA (50 μM) for 1 h. Cell lysates were prepared and assayed for IκBα phosphorylation by Western blot analysis. A representative blot of three independent experiments is shown. The bottom histogram shows the relative intensity of each band. * *p* < 0.05. (**C**) HAECs were pre-treated with Y-27632 (10 μM) before LPA (50 μM) stimulation for 1 h. Cell lysates were prepared and assayed for IκBα expression and RelA/p65 phosphorylation by Western blot analysis. A representative blot of three independent experiments is shown. The histograms show the relative intensity of each band. * *p* < 0.05. (**D**) HAECs were pre-treated with Y-27632 (10 μM) and then stimulated with LPA (50 μM) for 1 h. Cytoplasmic and nuclear extracts were prepared and assayed for nuclear translocation of RelA/p65 by Western blot analysis. A representative blot of three independent experiments is shown. The histograms show the relative intensity of each band. * *p* < 0.05. (**E**) HAECs were treated with LPA (50 μM) for 1 h. In a set of experiments, cells are pre-treated with Y-27632 (10 μM). Cells were fixed and stained with anti-RelA/p65 antibody (red) and Hoechst (blue) (magnification ×400). A representative photomicrograph of three independent experiments is shown. Scale bar, 10 μm. Data are expressed as means ± SEM.

NF-κB pathway is responsible for the transcriptional induction of inflammatory cytokines, chemokines and CAMs including MCP-1 and E-selectin [23,24]. Given ROCK’s ability to regulate activation of NF-κB signalling pathway [25,26,27,28,29], we investigated the mechanism underlying ROCK regulation of LPA-induced NF-κB activation. We first confirmed that NF-κB is involved in LPA-induced expression of E-selectin. As shown in Figure 2A, chemical inhibitor of NF-κB abolished MCP-1 and E-selectin induction by LPA. This data confirms that MCP-1 and E-selectin are strongly regulated by the NF-κB signalling. To investigate the effect of ROCK inhibition on phosphorylation and degradation of IκBα, well-characterized initial steps in NF-κB activation [30], we examined the kinetics of IκBα protein levels by Western blot analysis. Treatment with LPA caused a significant increase in phosphorylation of IκBα, which was reversed by ROCK inhibition (Figure 2B). Consistent with this observation, LPA-induced IκBα degradation was rescued and subsequent phosphorylation of RelA/p65 was decreased respectively by the treatment of Y-27632 (Figure 2C). These results indicate that ROCK signalling contributes to LPA-induced NF-κB activation through a mechanism that is dependent on IκBα degradation. Consistently, LPA increased nuclear-to-cytoplasmic ratio of RelA/p65 levels and this effect was attenuated by the inhibition of ROCK signalling (Figure 2D). Fluorescence microscopy (Figure 2E) also confirmed that RelA/p65 protein was predominantly localized in the cytoplasm under basal conditions and that exposure of endothelial cells to LPA resulted in cytoplasmic-to-nuclear translocation of RelA/p65 in a ROCK-dependent manner. These observations indicate that ROCK regulates NF-κB activation via phosphorylation of IκBα and thereby transactivates inflammatory mediators in endothelial cells.

### 2.3. ROCK2 is Required for LPA-Induced MCP-1 and E-Selectin Expression in HAECs

**Figure 3:**
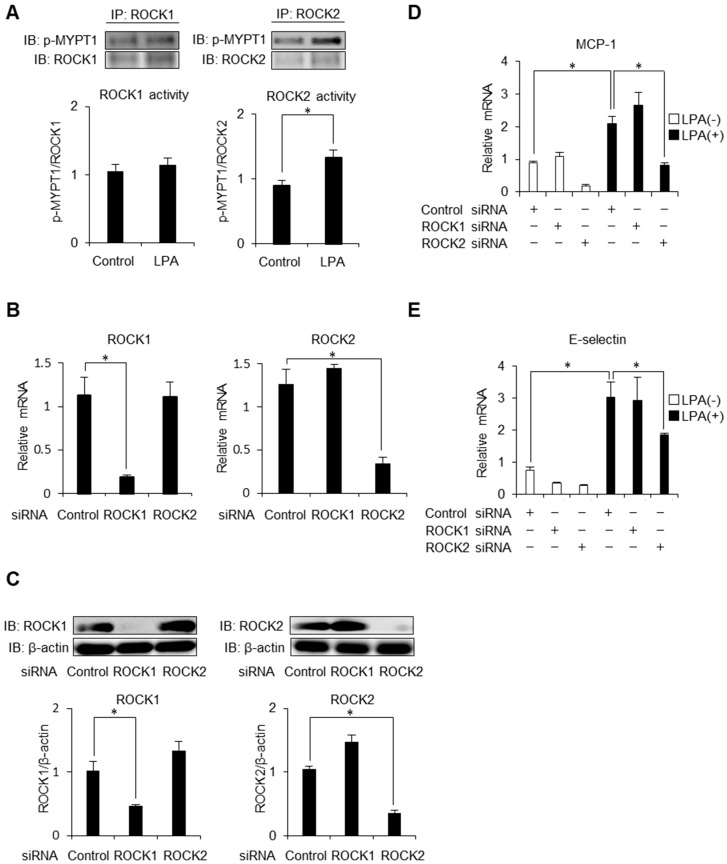
LPA-induced MCP-1 and E-selectin expression is possibly mediated via ROCK2 in HAECs. (**A**) HAECs were stimulated with LPA (50 μM) for 5 min. Cell lysates were subjected to immunoprecipitation analysis for detecting ROCK1 (left panel) or ROCK2 (right panel) activity. A representative blot of three independent experiments is shown. Each histogram shows the ratio of each band. * *p* < 0.05. (**B**) HAECs were treated with scrambled control siRNA or Rho-kinase isoform specific siRNA. Relative mRNA levels of ROCK1 (left panel) and ROCK2 (right panel) were analysed by quantitative real-time PCR (*n* = 3). * *p* < 0.05. (**C**) HAECs were treated with scrambled control siRNA or Rho-kinase isoform specific siRNA. Cell lysates were prepared and assayed for ROCK1 and ROCK2 by Western blot analysis. A representative blot of three independent experiments is shown. Each histogram shows the ratio of each band. * *p* < 0.05. (**D**,**E**) HAECs stimulated with LPA (50 μM) for 4h or 8 h were treated with scrambled control siRNA or Rho-kinase isoform specific siRNA and relative mRNA levels of MCP-1 (**D**) and E-selectin (**E**) were analysed by quantitative real-time PCR (*n* = 3). * *p* < 0.05. Data are expressed as means ± SEM.

ROCK has two isoforms, ROCK1 and ROCK2 [18]. In order to examine isoform-specific activities of ROCK, we first performed immunoprecipitation assay. Intriguingly, LPA selectively activated ROCK2 in endothelial cells (Figure 3A). We next knocked down ROCK1 and ROCK2 separately using siRNA duplexes to determine the role of isoform-dependent contribution on endothelial inflammation. By knocking down ROCK1 or ROCK2, cell shrinkage was observed, confirming the reduction of ROCK [14]. We observed sufficient knockdown efficiency by analysing relative mRNA levels and protein levels of ROCK1 as well as ROCK2. The mRNA and protein levels of ROCK1 or ROCK2 were significantly lower, with a compensatory greater level of ROCK2 or ROCK1, in HAECs with gene silencing (Figure 3B,C). As shown in Figure 3D,E, individual knockdown of ROCK1 had no effects on mRNA expressions of MCP-1 and E-selectin. In contrast, gene silencing of ROCK2 resulted in an attenuation of MCP-1 and E-selectin expressions, indicating that ROCK2 isoform but not ROCK1 is required for these inductions. These findings suggest that ROCK2 but not ROCK1 governs LPA-driven inflammatory reactions.

### 2.4. Endothelial ROCK2 Regulates Recruitment of Monocytic Cells

**Figure 4:**
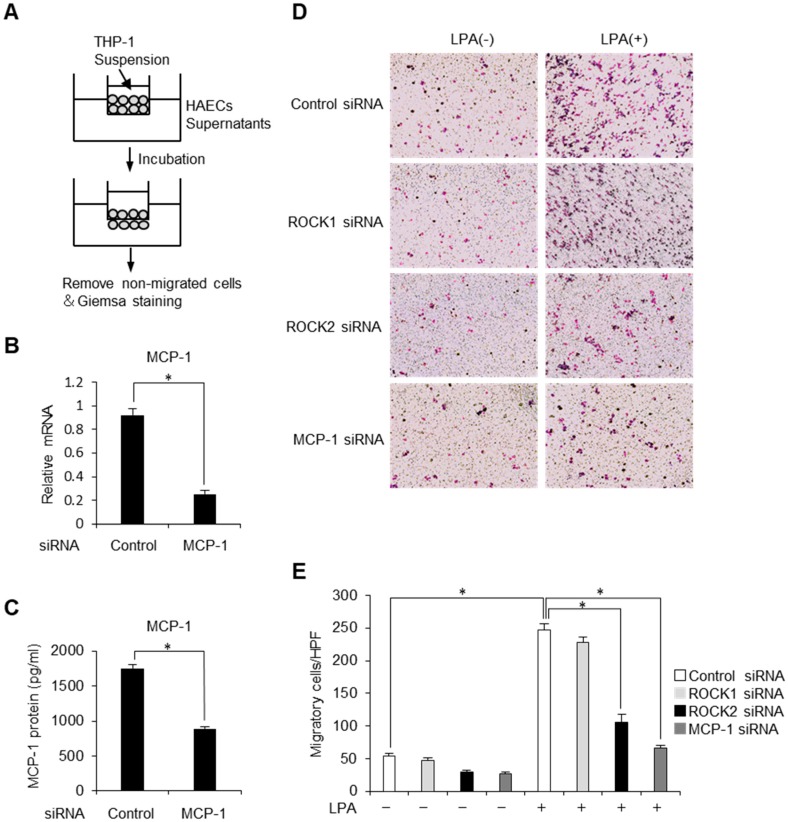
Endothelial ROCK2 regulates recruitment of monocytic cells. (**A**) The principle of migration assay. (**B**) HAECs were treated with scrambled control siRNA or MCP-1 siRNA. Relative mRNA levels of MCP-1 were analysed by quantitative real-time PCR (*n* = 3). * *p* < 0.05. (**C**) HAECs were treated with scrambled control siRNA or MCP-1 siRNA. Culture media were harvested and MCP-1 protein levels were analysed by ELISA (*n* = 3). * *p* < 0.05. (**D**,**E**) HAECs stimulated with LPA (50 μM) for 12 h were treated with scrambled control siRNA, Rho-kinase isoform specific siRNA or MCP-1 siRNA. The HAECs supernatants were then collected and assayed for their chemotactic activity on THP-1 cells through Transwell chemotaxis chamber. The migrated THP-1 cells were observed with the use of light microscopy (magnification ×100) (**D**) and the migrated cells were counted (**E**) (*n* = 3). * *p* < 0.05. Data are expressed as means ± SEM.

We next sought to understand the biological significance of ROCK2 in migration and adhesion of monocytes toward endothelial cells. To evaluate recruitment of THP-1, human monocyte lineage cells, across a chamber exposed to conditioned medium, we conducted chemotaxis assay (Figure 4A). First, we examined whether MCP-1 is involved in LPA-induced monocytic migration. By knocking down MCP-1, we observed sufficient knockdown efficiency by analysing relative mRNA levels and protein secretion of MCP-1 (Figure 4B,C). In the cells treated with control siRNA, the number of chemotactic cells to the lower chamber was significantly increased in the medium obtained from LPA-stimulated HAECs, which was greatly inhibited by gene silencing of MCP-1. While it was not affected by the treatment with ROCK1-specific siRNA, gene silencing of ROCK2 robustly inhibited LPA-induced monocyte chemotaxis (Figure 4D,E). These findings indicate that endothelial ROCK2 regulates MCP-1-induced recruitment of monocytic cells.

### 2.5. ROCK2 Controls Cell to Cell Adhesion in HAECs

**Figure 5:**
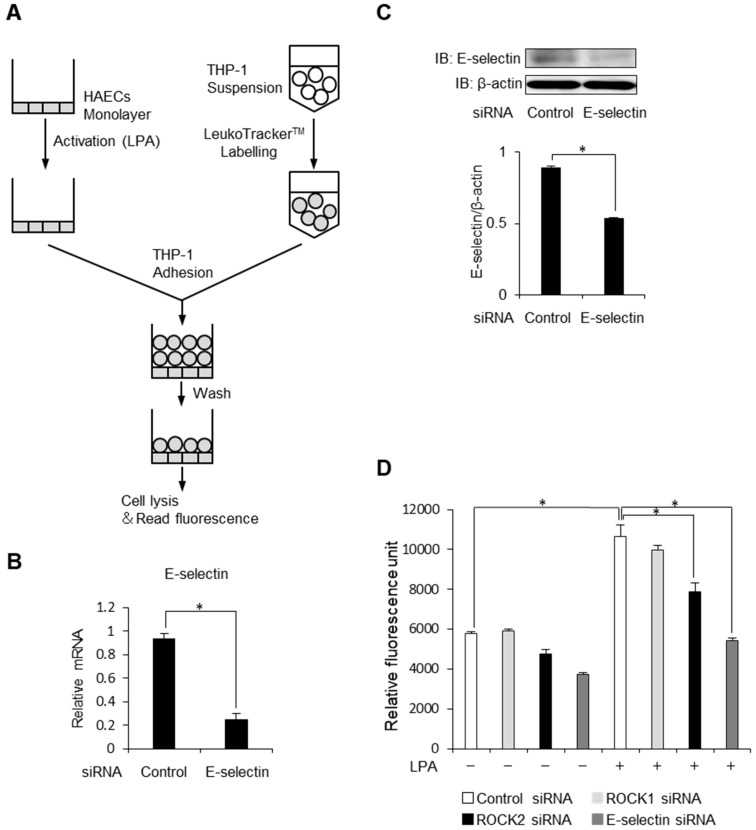
Endothelial ROCK2 regulates cell to cell adhesion. (**A**) The principle of adhesion assay. (**B**) HAECs were treated with scrambled control siRNA or E-selectin siRNA. Relative mRNA levels of E-selectin were analysed by quantitative real-time PCR (*n* = 3). * *p* < 0.05. (**C**) HAECs were treated with scrambled control siRNA or E-selectin siRNA. Cell lysates were prepared and assayed for E-selectin by Western blot analysis. A representative blot of three independent experiments is shown. The bottom histogram shows the ratio of each band. * *p* < 0.05. (**D**) HAECs stimulated with LPA (50 μM) for 8 h were treated with scrambled control siRNA or Rho-kinase isoform specific siRNA. Cell lysates were analysed for cell adhesion to THP-1 using adhesion assay. The bar graph shows the relative fluorescence unit of each sample (*n* = 3). * *p* < 0.05. Data are expressed as means ± SEM.

**Figure 6:**
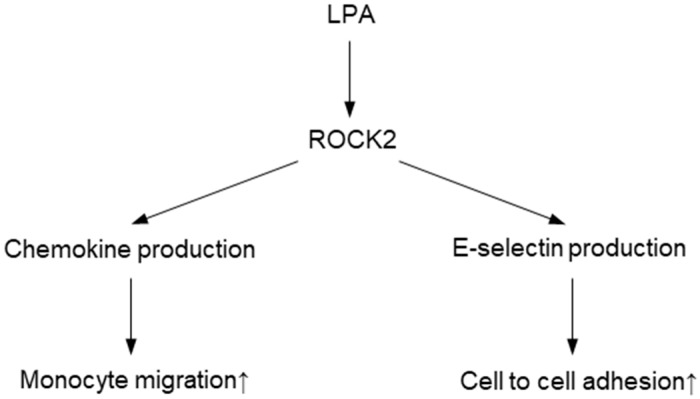
Mechanism of ROCK2-mediated cell to cell adhesion and monocyte migration in HAECs. In vascular endothelial cells, LPA induces selective activation ROCK2 and expression of chemokines and E-selectin. As a result, cell to cell adhesion and monocyte migration are increased.

Finally, we performed an in vitro adhesion assay using LeukoTracker^TM^ labelled THP-1 and monolayers of HAECs to evaluate functional significance of ROCK2-mediated E-selectin induction in endothelial cells (Figure 5A). First, we confirmed whether E-selectin takes part in LPA-induced cell to cell adhesion. Knocking down efficiency of E-selectin is shown in Figure 5B and 5C. In HAEC monolayer treated with control siRNA, stimulation of LPA increased the number of adherent cells, which was largely decreased by knocking down of E-selectin (Figure 5D). While individual knockdown of ROCK1 had no effects on the number of adherent cells, ROCK2 gene knockdown by RNA interference resulted in an attenuation of cell to cell adhesion. These findings indicate that endothelial ROCK2 regulates E-selectin-induced cell to cell adhesion. Taken together, our results suggest that ROCK2 but not ROCK1, regulates cell to cell recruitment and adhesion of monocytic cells in endothelial cells (Figure 6).

## 3. Discussion

Chemokine-driven transendothelial migration of monocytes and selectin-mediated adhesion toward endothelial cells are critical steps in the development of atherosclerosis. As such, it is important to identify a factor that regulates these processes to develop a novel therapeutic strategy against atherosclerosis.

ROCK is known to induce endothelial dysfunction by NF-κB activation. However, precise mechanisms underlying this observation have not been elucidated. Activation signals induce the phosphorylation of IκB by IκB kinase (IKK), which triggers the degradation of IκBα through the ubiquitin system, allowing free NF-κB RelA/p65 to translocate to the nucleus and activate transcription of target genes [31,32]. ROCK does not regulate NF-κB signalling pathway uniformly. Although our laboratory [17] and Anwar et al. showed that ROCK regulates thrombin-mediated p65 phosphorylation and IκBα phosphorylation in endothelial cells [33], the contribution of ROCK in LPA-mediated NF-κB activation has not been reported. Our group previously reported that ROCK regulates nuclear translocation of RelA/p65 via actin dynamics in mesangial cells without altering p65 phosphorylation [34]. Antoniellis et al. recently reported the possibility that RhoA, an upstream signalling molecule of ROCK, may regulate the NF-κB (p50) translocation in neutrophils [35]. These data suggest that ROCK regulates nuclear translocation of multiple NF-κB components. Therefore, further studies will be interesting to examine whether similar observations are observed in endothelial cells. In the present study, we found that ROCK mediates LPA-induced phosphorylation of IκBα as well as subsequent phosphorylation and nuclear translocation of p65 (Figure 2B–E). These observations suggest that the way of regulating NF-κB varies depending on the kinds of stimuli and types of cells.

The present study identifies ROCK2 as a key regulator of endothelial inflammation and illustrates its important role in atherogenic process. Different roles of ROCK1 and ROCK2 have been implicated because they cannot fully compensate for each other’s loss [36]. ROCK2 has been shown to be expressed in human vascular endothelial cells [37]. Shimada et al. reported the contribution of ROCK2 to the induction of ICAM-1 and VCAM-1, suggesting that ROCK2 functions as main isoform in endothelial inflammation [2]. However, it has not been elucidated whether ROCK2 is involved in regulating monocytic migration and adhesion toward endothelial cells. In the present study, we demonstrated for the first time that ROCK2 but not ROCK1 regulates these processes (Figure 4A–E, 5A–D). These findings enhanced the importance of ROCK2 in endothelial dysfunction. It has also been demonstrated that ROCK2 deficiency in bone marrow-derived cells showed substantially reduced lipid accumulation and atherosclerotic lesions in the LDL receptor-null mice and this was associated with decreased foam cell formation and increased cholesterol efflux in ROCK2 deficient macrophages [38]. Further, elegant study from Shimokawa Laboratory showed that ROCK2 in vascular smooth muscle cell (VSMC) contributes to the pathogenesis of cardiovascular diseases, including pulmonary arterial hypertension [39]. These observations elucidate ROCK2 as an important regulator of the inflammatory circuitry that governs the development of cardiovascular disease in various cell types (macrophages, VSMCs and endothelial cells) involved in the development of atherosclerosis.

Until recently, only one ROCK inhibitor has been approved for clinical use in Japan and China. Fasudil, a ROCK inhibitor, was clinically approved in 1995 in Japan for the prevention and treatment of cerebral vasospasm after surgery for subarachnoid haemorrhage [40]. Besides fasudil, more than 170 different ROCK inhibitors have been developed [41]. Recently, ripasudil was approved in Japan for the treatment of glaucoma and ocular hypertension [42]. Of note, SLx-2119, that has 100-fold more selectivity towards ROCK2 than ROCK1, has been suggested to be a potential drug for the treatment of ischemic stroke [43], autoimmune disease [44,45] and psoriasis [46]. To confirm our findings and its therapeutic significance in human, clinical studies of ROCK2 inhibitors in patients with atherosclerosis will be intriguing.

The present study has several limitations. First, we found that ROCK2 regulates monocytic migration and adhesion toward endothelial cells, which are essential steps in the development of atherosclerotic lesions. However, it remains unknown how ROCK2 affects other pathological machinery in endothelial cells (i.e., angiogenesis, cell death, hyperpermeability, impaired energy metabolism). Comprehensive microarray-based pathway analysis will be required to understand the importance of endothelial ROCK2. Second, our PCR array analysis showed increased expression of CXCL12 and demonstrated a trend toward to enhance mRNA expression of other chemokine ligands (e.g., CXCL10, 11) and receptors (e.g., CXCR1, 2) in endothelial cells treated with Rho-kinase inhibitor. CXCL12 has been shown to regulate the recruitment of smooth muscle progenitor cells and overproduction of CXCL12 may result in vascular remodelling [47]. Furthermore, CXCR1 and CXCR2 are crucial chemokine receptors for neutrophil recruitment during inflammation [48]. Considering the evidence that long-term oral treatment with Rho-kinase inhibitor markedly attenuated the accumulation of macrophage and the coronary lesion formation in a porcine model of atherosclerosis [49], we believe Rho-kinase inhibition is beneficial for the prevention of atherogenic changes: however, in order apply to ROCK2-targeted therapy in a clinical setting, further investigation in vivo will be required. Tissue-specific and inducible gene expression studies may offer additional insights into the spatial and temporal in vivo contribution of ROCK2 to endothelial dysfunction and provide further avenues of investigation. Third, because gene silencing of ROCK2 does not completely inhibit monocyte migration and cell adhesion in HAECs (Figure 4D,E and Figure 5D), there is a possibility that other signals also regulate them.

In conclusion, the current study suggested that ROCK2 mediates LPA-induced monocytic migration and adhesion to endothelial cells by attenuating NF-κB-dependent inductions of chemokines and CAMs. Our findings raise the possibility that targeting endothelial ROCK2 may be a feasible approach against atherosclerosis.

## 4. Materials and Methods

### 4.1. Reagents

ROCK1 antibody (Cat# sc-17794), ROCK2 antibody (Cat# sc-398519), β-actin antibody (Cat# sc-47778), Ku-70 antibody (Cat# sc-17789), myosin phosphatase target subunit 1 (MYPT1) antibody (H-130) (Cat# sc-25618), mouse anti-goat IgG-HRP (Cat# sc-2354), m-IgGκ BP-HRP (Cat# sc-516102), Protein A/G PLUS-Agarose Immunoprecipitation Reagent were purchased from Santa Cruz Biotechnology (Santa Cruz, CA, USA). Predesigned human small interfering RNA (siRNA) duplexes against ROCK1 (Cat# sc-29473), ROCK2 (Cat# sc-29474), MCP-1 (Cat# sc-43914), E-selectin (Cat# sc-29296) and control siRNA (Cat# sc-37007) also were purchased from Santa Cruz Biotechnology. IκBα (L35A5) antibody (Cat# 4814), phospho-RelA/p65 antibody (Ser536) (Cat# 3031) and RelA/p65 antibody (Cat# 8242) were from Cell Signalling Technology (Beverly, MA, USA). LPA was purchased from Cayman Chemical (Ann Arbor, MI, USA). Bay 11-7082 (Cat# B5556), a NF-κB inhibitor, was purchased from Sigma-Aldrich (St. Louis, MO, USA). Y-27632 (Cat# 030-24021), a ROCK inhibitor, was obtained from Wako (Osaka, Japan). E-selectin antibody (Cat# ab18981) was purchased from Abcam (Cambridge, UK). Phospho-MYPT1 antibody (Thr850) (Cat# 36-003) was from EMD Millipore corp (Burlington, MA, USA). Goat anti-Rabbit IgG (H+L) secondary antibody (Cat# 32460) was from Thermo Fisher Scientific (Rockford, IL, USA). Anti-IgG (H+L chain) mouse pAb-HRP (Cat# 330) was from MBL (Nagoya, Japan).

### 4.2. Cell Culture

Human aortic endothelial cells (HAECs) (Cat# C-12271) and THP-1 (Cat# TIB202) were purchased from PromoCell (Heidelberg, Germany) and ATCC (Manassas, VA, USA) respectively but were not further authenticated after purchase. Testing for mycoplasma contamination has not been performed. All cells were grown at 37 °C in humidified air containing 5% (*v*/*v*) CO_2_. For experiments with inhibitors, HAECs were incubated with the indicated concentration of agents for 30 min before stimulation.

### 4.3. PCR Array

To explore inflammatory genes regulated by ROCK in vascular endothelial cells, we analysed HAECs gene expression by using Human Inflammatory Cytokines & Receptors RT2 Profiler PCR Array (Qiagen, Hilden, Germany) after HAECs had been treated with LPA (50 μM) for 8 h with or without gene silencing of ROCK2. Gene expression heat map was displayed using MeV, a Java tool for genomic data analysis.

### 4.4. Enzyme-Linked Immunosorbent Assay (ELISA)

The concentration of MCP-1 in the conditioned medium was determined with a human CCL2/MCP-1 Quantikine ELISA kit (R&D Systems, Minneapolis, MN, USA) according to the manufacturer’s instructions. In brief, we added diluted culture medium to 96-well microplates coated with polyclonal antibody that detects human MCP-1 protein. After the incubation and subsequent washes, enzyme-linked polyclonal antibody for MCP-1 was added. After another incubation and washes, we added colour reagents. The signalling intensity was detected by microplate reader at 450 nm/540 nm.

### 4.5. RNA Isolation and Quantitative Real-Time PCR

Total RNA was isolated from HAECs with TRIzol reagent (Invitrogen) followed by chloroform-isopropanol extraction and ethanol precipitation and 1 μg of total RNA was reverse-transcribed using the Prime Script RT reagent Kit (Takara Bio, Otsu, Japan). To evaluate the mRNA expression of MCP-1 and E-selectin, we performed quantitative real-time PCR analysis by the Thermal Cycler Dice Real Time System TP800 (Takara Bio) by use of SYBR Green I fluorescence signals. Primers used for PCR were as follows: human E-selectin, 5′-AGAGTGGAGCCTGGTCTTACA-3′ (forward) and 5′-CCTTTGCTGACAATAAGCACTGG-3′ (reverse); human MCP-1, 5′-CAGCCAGATGCAATCAATGCC-3′ (forward) and 5′-TGGAATCCTGAACCCACTTCT-3′ (reverse); human β-actin, 5′-CATGTACGTTGCTATCCAGGC-3′ (forward) and 5′-CTCCTTAATGTCACGCACGAT-3′ (reverse); human ROCK1, 5′-AACATGCTGCTGGATAAATCTGG-3′ (forward) and 5′-TGTATCACATCGTACCATGCCT-3′ (reverse); human ROCK2, 5′-TCAGAGGTCTACAGATGAAGGC-3′ (forward) and 5′-CCAGGGGCTATTGGCAAAGG-3′ (reverse).

### 4.6. Western Blot Analysis

Whole cell lysates were extracted by RIPA buffer. Nuclear extracts were prepared using NE-PER Nuclear and Cytoplasmic Extraction Reagents (Pierce, Rockford, IL, USA) as directed. Equal amounts of protein samples were loaded onto SDS-PAGE gels, electrophoresed and transferred onto nitrocellulose membranes (Invitrogen). After blocking in non-fat milk, we incubated membranes with primary antibodies and then incubated with the corresponding secondary horseradish peroxidase-conjugated antibody. The signal intensity was measured by a LAS-4000 mini Luminescent Image Analyzer (FUJIFILM, Tokyo, Japan).

### 4.7. Transfection and Reporter Gene Assay

The plasmid pGL3-ELAM-LUC (plasmid#13029, Addgene, Watertown, MA, USA), containing five copies of consensus E-selectin sequences linked to the luciferase gene, was transfected into HAECs using FuGENE 6 reagent (Roche Diagnostics, Mannheim, Germany) according to the manufacturer’s instructions. A total of 2 μg plasmid DNA was used per well. Twenty-four hours after the transfection, cells were treated with inhibitors before stimulation with LPA. E-selectin activity was determined using the Dual-Luciferase H Reporter Assay System (Promega, Madison, WI, USA).

### 4.8. Immunocytochemistry

HAECs grown on glass coverslips were washed with PBS and fixed with 10% (*v*/*v*) formalin for 15 min. For detection of E-selectin and RelA/p65, we permeabilized fixed cells with 0.2% (*v*/*v*) Triton X-100 and blocked with PBS containing 10% (*v*/*v*) normal goat serum. The cells were then incubated with an anti-E-selectin antibody (1:200) and anti-RelA/p65 antibody (1:200) at 4 °C overnight. After washing, cells were incubated with Alexa Fluor 532-conjugated secondary (1:100; Molecular Probes, Eugene, OR, USA) for 1 h. Nuclei were visualized using Hoechst dye. Images were observed on a BZ-9000 fluorescence microscope (Keyence, Osaka, Japan) using BZ-II; analysis application (Keyence, Osaka, Japan).

### 4.9. RNA Interference

HAECs at ~50% confluence were transfected with control siRNA (negative control), ROCK1 siRNA, ROCK2 siRNA, MCP-1 siRNA or E-selectin siRNA using Lipofectamine reagent (Invitrogen, Carlsbad, CA, USA) according to the manufacturer’s instructions.

### 4.10. Immunoprecipitation

Whole-cell lysates (500 μg) of HAECs were prepared with RIPA buffer. The immunoprecipitation assay was performed using Protein A/G PLUS-Agarose (Santa Cruz Biotechnology) with ROCK1 or ROCK2 antibody according to the manufacturer’s protocol. Activity of ROCK1 or ROCK2 was assessed by western blot analysis using Phospho-myosin phosphatase target subunit 1 (MYPT1) antibody (Thr850). In this assay, the expression levels of ROCK1 and ROCK2 were identified based on the corresponding molecular weight without loading positive control samples.

### 4.11. Chemotaxis Assay

THP-1 monocyte chemotaxis was evaluated in a 24-well Transwell chemotaxis chamber separated by a 5 μm pore size polycarbonate membrane filter (Costar, Cambridge, MA, USA). HAECs grown to 50% confluence were transfected with control siRNA, ROCK1 siRNA, ROCK2 siRNA or MCP-1 siRNA as described above. After 48 h, cells were stimulated with LPA (50 μM) for 12 h. Then we added an aliquot of the culture medium to the lower chamber. An aliquot of THP-1 monocyte suspension (1.0 × 10^5^ cells/100 μL) was placed in the upper chamber. The dish was incubated at 37 °C for 2 h to promote transmigration of monocytes. The migrated cells that adhered to the lower surface of the membrane were stained with Giemsa’s solution and counted on four randomly-selected high power fields.

### 4.12. Adhesion Assay

The THP-1 adhesion to monolayer of HAECs was determined using a CytoSelect Adhesion Assay Kit (Cell Biolabs, San Diego, CA, USA) according to manufacturer’s instructions. At 96-well plates, HAECs grown to 50% confluence were transfected with control siRNA, ROCK1 siRNA, ROCK2 siRNA or E-selectin siRNA as described above. After 48 h, cells were stimulated with LPA (50 μM) for 8 h. After LPA treatment, THP-1 cells (1.0 × 10^6^ cells/mL) were labelled with LeukoTracker, added to the wells and incubated for 1 h at 37 °C. The HAECs were washed to remove the non-adherent THP-1 cells, lysed and the fluorescence was measured with a spectrofluorometer (Promega) at 480 nm/520 nm. The experiment was repeated three times with duplicate assay and the results were expressed as relative fluorescence unit.

### 4.13. Statistical Analysis

All in vitro data are from three independent experiments. Data are expressed as means ± SEM. Comparison of groups was performed using analysis of variance and Bonferroni’s post hoc correction. A value of *p* < 0.05 was considered statistically significant.

## Abbreviations

ROCK	Rho-kinase
LPA	lysophosphatidic acid
CAMs	chemokines and cell adhesion molecules
MCP-1	monocyte chemoattractant protein-1
HAECs	human aortic endothelial cells
VCAM-1	vascular cell adhesion molecule-1
ICAM-1	intracellular adhesion molecule-1
MYPT1	myosin phosphatase target subunit 1
siRNA	small interfering RNA
IKK	IκB kinase
VSMC	vascular smooth muscle cell
